# Comparison of postoperative pain between laparoscopic and robot-assisted partial nephrectomies for renal tumors: A propensity score matching analysis

**DOI:** 10.1097/MD.0000000000007581

**Published:** 2017-07-21

**Authors:** Seok-Joon Jin, Jun-Young Park, Doo-Hwan Kim, Syn-Hae Yoon, Eunkyul Kim, Jai-Hyun Hwang, Cheryn Song, Young-Kug Kim

**Affiliations:** a Department of Anesthesiology and Pain Medicine; b Department of Urology, Asan Medical Center, University of Ulsan College of Medicine, Seoul, Republic of Korea.

**Keywords:** laparoscopic partial nephrectomy, postoperative pain, renal tumor, robot-assisted partial nephrectomy

## Abstract

Robot-assisted partial nephrectomy (RAPN) has emerged as an alternative to laparoscopic partial nephrectomy (LPN) for removal of renal tumors. Several advantages of robotic surgery have been reported, but there is no comparative study on postoperative pain between the 2 techniques. Therefore, we compared the postoperative numerical rating scale (NRS) of pain intensity between patients who underwent LPN and those who underwent RAPN.

We included 705 patients who underwent either LPN (n = 200) or RAPN (n = 505) for renal tumors between January 2000 and September 2016. After 1:1 propensity score matching, the final analysis included 142 patients each in the LPN and RAPN groups. The primary endpoint was postoperative NRS of pain intensity. The secondary endpoints were opioid requirement, opioid-related complications, and duration of hospital stay.

Preoperative and intraoperative values of propensity score matched patients (n = 284) were not significantly different between the LPN and RAPN groups. There was no significant difference in NRS of pain intensity between the 2 groups. Opioid requirement was different between the 2 groups on postoperative day (POD) 0 (12.4 vs 11.3 mg of morphine-equivalent dose), but not from POD 1 to POD 4. The incidence of opioid-related complications and duration of hospital stay were not significantly different between the 2 groups.

Postoperative pain was not significantly different between patients who underwent RAPN and those who underwent LPN. This result provides a potentially useful knowledge of postoperative pain characteristics in RAPN and LPN.

## 1 Introduction

Laparoscopic partial nephrectomy (LPN) was first adopted to treat small and peripheral renal tumors in 1993; since then, LPN has been widely implemented in clinical setting. However, LPN is limited in that the procedure requires a fair amount of surgeon experience. Major technical obstacles issues during an LPN includes aligning favorable angles for tumor excision and sutured repair, achieving hemostasis, redressing of the collecting system, and reconstructing the parenchymal defect within a finite ischemia time. Recent advances in robotic surgery have led several studies to compare LPN and robot-assisted partial nephrectomy (RAPN).^[1–3]^ These studies have reported that there were no significant differences between RAPN and LPN regarding postoperative outcomes. Nevertheless, RAPN has benefited from advancements in the robotic surgical system, including a greater range of motion, optically magnified imaging, and better precision of control, thereby resulting in reduced total operation time and less amount of experience required by the surgeon.^[4,5]^

Inadequate management of postoperative pain leads to a significantly higher intensity of pain up to 24 hours after surgery and increased requirements for analgesics, which may be associated with postoperative complications.^[6–8]^ Till date, however, no study has evaluated the differences in postoperative pain intensities between patients who have undergone LPN and those who have undergone RAPN.

In the current study, we compared the Numerical Rating Scale (NRS) of pain intensity in patients with renal tumors who underwent LPN or RAPN using a propensity score matching analysis. We also compared opioid requirements, opioid-related complications, and duration of hospital stay between the 2 modes of surgery.

## 2 Materials and methods

### 2.1 Patient characteristics

The Institutional Review Board of Asan Medical Center, Seoul, Republic of Korea approved this study (approval number: 2016–1045). We enrolled patients who underwent either LPN or RAPN for renal tumors between January 2000 and September 2016. Exclusion criteria were as follows: age of < 18 or ≥ 80 years, history of preoperative opioid use, incomplete data from medical records, use of retroperitoneal approach, conversion to an open partial nephrectomy, and combined operations.

### 2.2 General anesthesia

After patient monitoring was established, the anesthetic induction was performed with an intravenous bolus administration of thiopental or propofol and rocuronium or vecuronium. After endotracheal intubation, general anesthesia was maintained using sevoflurane, desflurane, or propofol. Target-controlled infusion of remifentanil was routinely used in combination with propofol, and was performed according to the preference of the anesthesiologist when used with an inhalation agent. Mechanical ventilation was titrated to achieve the end-tidal carbon dioxide concentration between 35 and 40 mmHg. Intravenous patient-controlled analgesia with fentanyl was used for pain control except for those who refused it. The physician made the decision to use opioids during the postoperative period if the NRS of pain intensity was greater than 6 (0 was defined as no pain, and 10 was defined as the worst pain ever experienced).^[9,10]^

### 2.3 Surgical procedure

LPN was performed as previously described.^[11]^ Briefly, three 12-mm trocars for the camera and working channels and one 5-mm trocar for assistant use were placed in the abdomen. Another 12-mm flexible trocar was used when Satinsky clamp was used for pedicle clamping. After total renal artery clamping, the tumor, including margins, was excised using cold scissors. Hemostatic sutures and collecting system repair were carried out as needed by using continuous running sutures. After complete renorrhaphy, the kidneys were reperfused in all cases. The surgical procedure was similar for RAPN except for the use of the da Vinci surgical system. Two 12-mm trocars for the camera and assistant use and three 8-mm trocars for robot instruments were placed in the abdomen, and the robot was docked accordingly. Trocar sites larger than 5 mm (8 mm robot, 12 mm laparoscopy) were closed with 2–0 polyglactin (Vicryl) interrupted suture for fascia, and 3–0 polyglecaprone (Monocryl) for subcutaneous layer. Only the subcutaneous layer was closed for the 5 mm trocar site, and all skin wounds were closed with topical adhesives (Dermabond). Excision and repair were performed in a similar manner for both procedures.

### 2.4 Clinical data collection

Demographic data, preoperative laboratory values, intraoperative variables, NRS of pain intensity, and opioid requirements until postoperative day (POD) 4 were collected. Reports of opioid-related complications and hospital stay were also obtained from the electronic medical record system. Demographic data included sex, age, body mass index, the American Society of Anesthesiologists (ASA) physical status class, and comorbidities. Preoperative laboratory values included hemoglobin, platelets, albumin, creatinine, and prothrombin time. Intraoperative variables included operators, anesthetic agents, use of remifentanil, and operation time.

### 2.5 Primary and secondary endpoints

Primary endpoint was the comparison of NRS of pain intensity between the LPN and RAPN groups on PODs 0, 1, 2, 3, and 4. Secondary endpoints were comparisons of opioid requirements, opioid-related complications, and duration of hospital stay between the 2 groups. All opioids and analgesics were converted to morphine-equivalent doses using morphine sulfate equivalents.^[12–14]^ Opioid-related complications included nausea, vomiting, dizziness, urticaria, constipation, headache, and sedation. Duration of hospital stay was determined from the day after either LPN or RAPN.

### 2.6 Statistical analysis

Data are expressed as mean ± standard deviation or number (percentage) as appropriate. Data variables included in this study were compared between LPN and RAPN groups using *χ*^*2*^ test or Fisher exact test for categorical variables and Student *t* test or Mann–Whitney *U* test for continuous variables. We performed multiple logistic regression analysis to determine the propensity score using the following 15 variables: sex, age, body mass index, ASA physical status class, diabetes mellitus, hypertension, preoperative laboratory values (hemoglobin, platelet, albumin, creatinine, and prothrombin time), operators, anesthetic agents, use of remifentanil, and operation time (Table 1). Propensity score matching was performed by Greedy matching using a caliper of 0.1 standard deviations of the logit of the propensity score. Model calibration was assessed using Hosmer–Lemeshow statistics (*χ*^*2*^ = 4.952; *df* = 18; *P* = .999). After performing 1:1 propensity score matching, continuous variables were compared using paired *t* test or Wilcoxon signed-rank test as appropriate. Categorical variables were compared using McNemar's test or marginal homogeneity test as appropriate. In all analyses, *P* < .05 was considered statistically significant. Statistical analysis was conducted using R (version 3.1.2; R Foundation for Statistical Computing, Vienna, Austria) and SPSS for Windows (version 23.0.0; IBM Corporation, Chicago, IL).

**Table 1:**
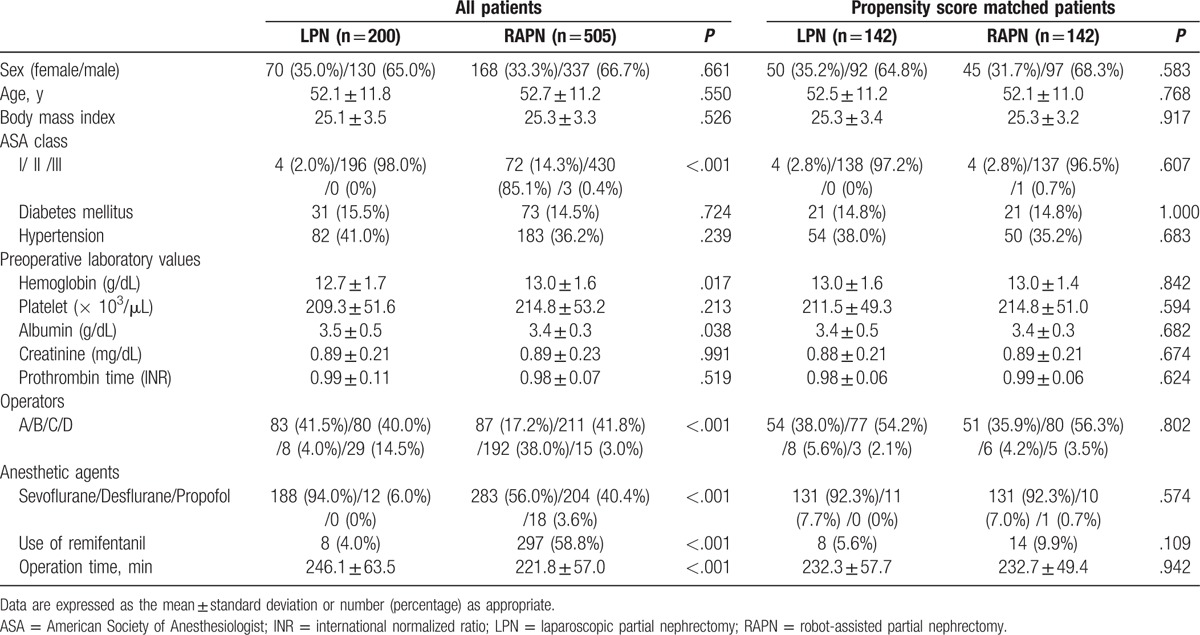
Preoperative and intraoperative variables.

## 3 Results

**Figure 1:**
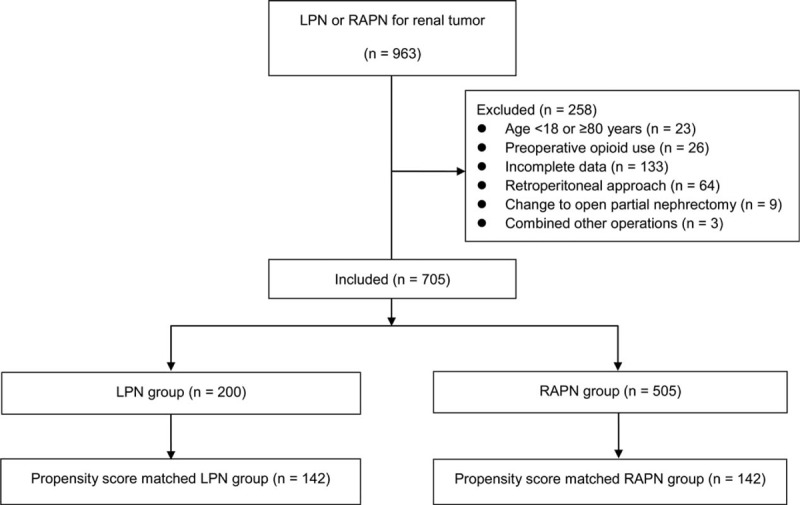
Study flow chart. LPN = laparoscopic partial nephrectomy; RAPN = robot-assisted partial nephrectomy.

Medical records of 963 patients who underwent either LPN or RAPN for renal tumors between January 2000 and September 2016 were reviewed. We excluded patients who met the following criteria: aged < 18 or ≥ 80 years at the time of surgery (n = 23); history of preoperative opioid use (n = 26); incomplete medical data (n = 133); use of retroperitoneal approach (n = 64); operation change to open partial nephrectomy (n = 9); and combined other operations (n = 3). Thus, 705 patients were included (200 in the LPN group, 505 in the RAPN group) (Fig. 1).

None of the patients included in this analysis have used opioids before LPN or RAPN. No patients underwent lymph node dissection during surgery. Neither chemotherapy nor radiotherapy was performed during the perioperative period.

Preoperative and intraoperative values of all patients (n = 705) and propensity score matched patients (n = 284) are listed in Table 1. Before the propensity score matching analysis, the 2 groups showed significant differences in ASA class, preoperative hemoglobin and albumin levels, operators, anesthetic agents, use of remifentanil, and operation time. After the propensity score matching analysis, there were no significant differences in these variables between the 2 groups (Tables 1 and 2).

**Table 2:**
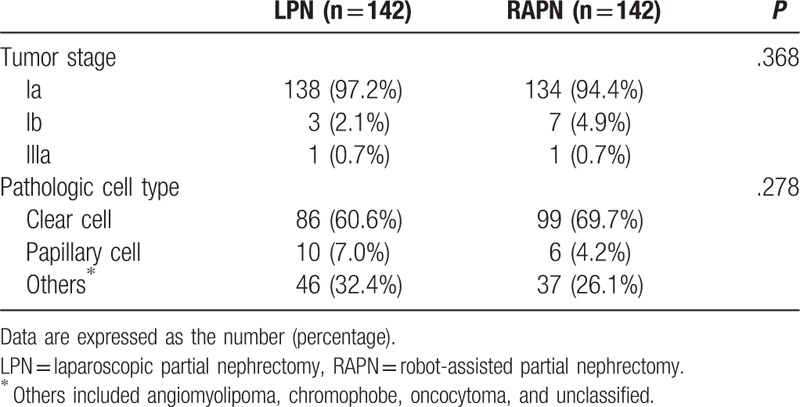
Tumor characteristics after propensity score matching.

**Figure 2:**
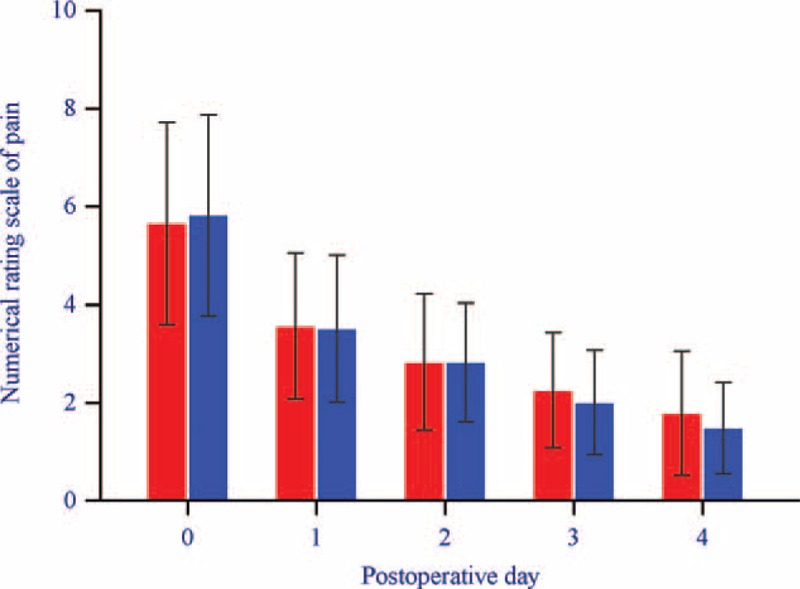
Numerical rating scales of pain intensity on postoperative days 0, 1, 2, 3, and 4 in LPN group (red box) and RAPN group (blue box). There were no significant differences in the numerical rating scales of pain intensity between the 2 groups at any time point. The upper borders of the box and error bars of each group represent the means and standard deviations, respectively. LPN = laparoscopic partial nephrectomy; RAPN = robot-assisted partial nephrectomy.

Postoperative NRSs of pain intensity were not statistically different between the LPN and RAPN groups (at POD 0, 5.7 vs 5.9, *P* = .443; at POD 1, 3.6 vs 3.5, *P* = .640; at POD 2, 2.8 vs 2.8, *P* = .680; at POD 3, 2.3 vs 2.0, *P* = .116; and at POD 4, 1.8 vs 1.5, *P* = .102) (Fig. 2). There was a significant difference in the opioid requirement at (morphine-equivalent dose) POD 0 between the LPN and RAPN groups (12.4 vs 11.3 mg, *P* = .004). There was no significant difference in the opioid requirements (morphine-equivalent doses) between the LPN and RAPN groups from POD 1 to POD 4 (at POD 1, 2.6 vs 2.8 mg, *P* = .435; at POD 2, 2.0 vs 2.5 mg, *P* = .201; at POD 3, 1.6 vs 1.5 mg, *P* = .599; and at POD 4, 1.3 vs 1.2 mg, *P* = .590). Incidence rates of opioid-related complications, including nausea, vomiting, dizziness, urticaria, constipation, headache, and sedation, were not significantly different between the LPN and RAPN groups (6.3% vs 10.6%, *P* = .307). Duration of hospital stay was also not significantly different between the LPN and RAPN groups (6.5 vs 6.4 days, *P* = .372).

## 4 Discussion

Because of technical differences between LPN and RAPN, we expected to observe the difference in postoperative pain intensity between the 2 modes of surgery. However, we found that the postoperative pain was not significantly different between patients who underwent LPN and those who underwent RAPN. In addition, there were no significant differences in opioid requirements between the 2 groups from POD 1 to POD 4. The incidence of opioid-related complications and the duration of hospital stay were similar between the 2 groups as well.

Previous studies have reported that there is no statistically significant difference in pain intensity after robot-assisted and laparoscopic surgery for gynecologic procedures.^[15,16]^ El Hachem et al^[15]^ showed that in gynecological surgeries, the pain intensity at POD 0 to POD 2 were not significantly different between a robotic and laparoscopic surgery. The opioid requirements were similar to our results, except that the opioid requirement at POD 0 was not significantly different between the 2 groups.^[15]^ Zechmeister et al^[16]^ also reported that they observed no differences in pain intensity and postoperative analgesic use between robot-assisted and laparoscopic gynecologic surgery. On the other hand, several studies have reported that laparoscopic surgery may be more painful because of the greater abdominal wall injury than robotic surgery. As a possible mechanism for the reduced pain after robotic hysterectomy, Chiu et al^[17]^ explain that the chance of using the abdominal wall for leverage is decreased because the robotic arms pivot at the port sites and move/rotate around a fixed remote center-of-motion, thereby decreasing mechanical injury at the abdominal wall. Accordingly, Martino et al^[18]^ showed that patients with endometrial cancer who underwent robot-assisted hysterectomies required a fewer analgesics and had less severe initial postoperative pain than did those who underwent laparoscopic surgery. The authors also suggested that the stability of the trocars used during robot-assisted hysterectomy likely reduced the degree of trauma to the abdominal wall, pain intensity, and opioid requirements. These controversial results concerning the postoperative pain intensity and opioid requirement between laparoscopic surgery and robotic surgery may partly be explained by differences in the targets of operation (urologic vs gynecologic), criteria of opioid administration, and surgeon's experiences.

We observed that opioid-related complications did not significantly differ between LPN and RAPN. Common side effects after opioid administration are nausea, vomiting, constipation, dizziness, physical dependence, sedation, and respiratory depression.^[19]^ In the current study, complications after opioid administration included nausea, vomiting, dizziness, urticaria, constipation, and sedation. All patients who experienced complications completely recovered without any severe complications.

We also found that postoperative hospital stay did not significantly differ between the 2 groups. Ellison et al^[20]^ reported that patients who undergo LPNs have shorter hospital stay durations compared with those who undergo RAPNs; however, pooled meta-analysis found no significant differences between the 2 surgeries.^[1]^ The reason for the absence of significant difference might be that the patients were generally discharged between POD 5 and POD 7 if there were no significant postoperative complications, such as postoperative bleeding.^[21]^

A limitation of this study is that this was a retrospective analysis. However, this study was conducted with a relatively large population. Furthermore, we performed propensity score matching analysis to control the various confounding variables. We included the surgeon's experiences, which may significantly influence postoperative pain.^[1]^ We believe that propensity score matching analysis for controlling the type of operators is necessary to accurately compare the effects of LPN or RAPN on postoperative pain.

In conclusion, patients who underwent an RAPN had similar postoperative pain intensity to those who underwent an LPN. Our results provide potentially useful information of postoperative pain characteristics in the 2 surgical methods.
